# Post-anastomotic venous stenosis after Optiflow deployment: An unexpected outcome

**DOI:** 10.1177/2050313X19851002

**Published:** 2019-05-30

**Authors:** Ziad Al Adas, George Haddad, Bhavin C Patel, Lalathaksha Kumbar, Baha Al-Abid, Praveen Balraj, Loay S Kabbani

**Affiliations:** 1 Department of Vascular Surgery, Henry Ford Hospital, Detroit, MI, USA; 2 Department of Nephrology, Henry Ford Hospital, Detroit, MI, USA

**Keywords:** Arteriovenous fistula, dialysis, Optiflow device, vascular graft stenosis

## Abstract

Arteriovenous fistula failure represents a major cause of hospitalization and a significant economic burden for end-stage renal disease patients on hemodialysis. The Optiflow (Bioconnect Systems Inc., Ambler, PA) is a new device developed to improve arteriovenous fistula outcomes and decrease failure rates by reducing the risk of stenosis and improving maturation rates. This case report describes a 50-year-old male with hypertensive nephropathy on dialysis who had multiple arteriovenous fistula failures in the past. He was scheduled to undergo brachiocephalic fistula construction using the Optiflow device. After 8 months of use, the new fistula developed a peri-anastomotic venous stenosis, just distal to the Optiflow device. To our knowledge, this is the first time such a complication has been reported.

## Introduction

**Figure 1.**
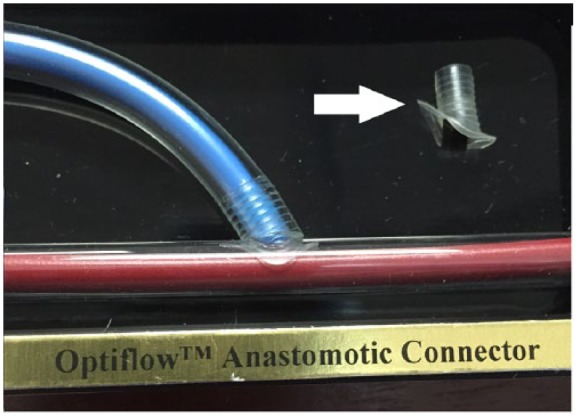
A model of the Optiflow device.

Arteriovenous (AV) fistulae have been reported to be superior to AV grafts and to central venous catheters for hemodialysis access, in terms of long-term function and reduced complication rates.^1^ Peri-anastomotic stenosis is one cause of fistula failure, occurring as a result of hemodynamic stress or trauma from poor surgical technique.^2,3^ Surgical experience has been proven to be a major determinant of AV fistula outcome; the Dialysis Outcomes and Practice Patterns Study showed that there was a 34% drop in the risk of early fistula failure if surgeons performed more than 25 fistulae during their training.^4^ Optiflow (Bioconnect Systems Inc., Ambler, PA) is a novel sutureless anastomotic connector, made from non-thrombogenic siliconized polyurethane (Figure 1). It aims at increasing AV fistula maturation rates through standardizing surgical technique and improving blood flow hemodynamics at the AV anastomosis. By providing a sutureless anastomosis and a fixed outflow tract that protects it from shear stress, the likelihood of intimal hyperplasia is reduced. Several animal and pilot human studies have proven the safety and efficacy of the Optiflow device in decreasing the incidence of venous luminal stenosis and improving maturation rates. In this case report, we present a patient who underwent Optiflow AV fistula placement and developed a post-anastomotic venous stenosis—an unpredictable outcome.

## Case description

A 50-year-old male, with end-stage renal disease (ESRD) secondary to hypertensive nephropathy, was selected to undergo creation of an AV fistula. He had undergone a radiocephalic fistula in his right arm that did not mature. After 6 months of hemodialysis via two tunneled cuffed dialysis catheters (one in the right internal jugular vein and another in the left), the patient presented to our institution for further evaluation. A venogram showed thrombosis of his radiocephalic fistula and no central venous obstruction. An ultrasound study revealed a cephalic vein that was 4.8 mm × 4.3 mm in diameter with no wall thickening. The brachial artery measured 5 mm × 5.3 mm with no wall thickening or calcification. He had triphasic waveforms in his brachial artery. The patient was briefed about his various vascular access options, and he agreed to proceed with a right brachiocephalic fistula construction using the Optiflow device. After discussing all the advantages and disadvantages, he signed the informed consent to be part of the OPEN (*O*ptiflow *P*at*E*ncy and Maturatio*N*) study.

**Figure 2.**
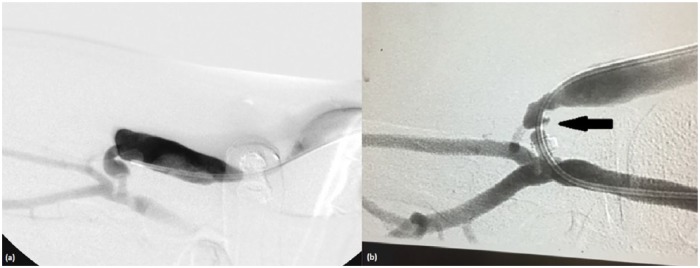
(a) Fistulogram after the placement of the Optiflow device. (b) Pre-dilation fistulogram showing the stenosis just distal to the Optiflow device.

The Optiflow device was inserted as per the manufacturer’s instructions. A 7-cm-long oblique incision was made in the antecubital fossa. The cephalic vein was mobilized and ligated distally. It was then moved to the brachial artery in a smooth line without tension or kinking. Before clamping the artery, the patient received 5000 U of heparin. The brachial artery was then clamped and an incision was made in the artery in the same plane and direction as the vein take-off angle. A vascular punch was used to make a circular arteriotomy 4 mm in diameter. The flanges of the Optiflow device were inserted into the artery using custom-made forceps. The vein was then attached to the Optiflow device. Four tacking stitches were placed in the adventitia between the artery and the vein. The subcutaneous tissues were closed using 3-0 Vicryl, and staples were used for skin closure. The operation was uneventful and a palpable thrill was noted at the anastomosis site after the operation (fistulogram—Figure 2(a)).

The new brachiocephalic fistula was cleared to be used for hemodialysis 5 weeks later. Seven weeks after the operation, the left internal jugular vein tunneled, cuffed dialysis catheter was removed, and hemodialysis was resumed via the right brachiocephalic fistula. A follow-up fistulogram conducted 9 weeks after the procedure showed no stenosis and good flow in the fistula and peri-anastomotic regions.

**Figure 3.**
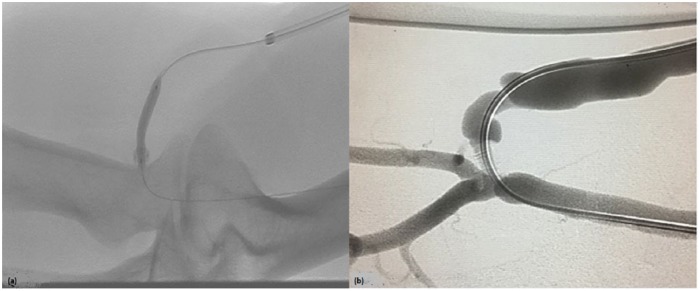
(a) Balloon angioplasty of the stenosis. (b) Post-angioplasty fistulogram showing no residual stenosis.

Eight months after the procedure, the patient was referred to us due to poor arterial blood flow during hemodialysis. A duplex ultrasound revealed increased flow velocity at the anastomosis site and decreased flow volume in the fistula. A venogram showed a 90% stenosis in the juxta-anastomotic area with aneurysmal dilation at this level (Figure 2(b)). Angioplasty could not be performed due to failure to cross the device in the outpatient vascular access center. A tunneled catheter was inserted. Four weeks later, the patient was taken to the hybrid operating room where the lesion was crossed via a retrograde approach using a 0.14 command ES (Abbott Laboratories, Abbott Park, IL). The patient underwent balloon angioplasty of the peri-anastomotic stenosis using a 4-mm AngioSculpt balloon (Angioscore Inc., Fremont, CA), followed by a 6-mm drug-coated Lutonix balloon (Bard Lutonix Inc., New Hope, MN) and leaving minimal residual stenosis (Figure 3(a) and (b)). Following the procedure, the patient resumed hemodialysis using the fistula after that.

Sixteen months following the Optiflow procedure (or 8 months post-angioplasty), the patient presented with fistula thrombosis for which thrombolysis and angioplasty were performed using the drug-coated balloon Admiral (Medtronic Inc, Santa Rosa, CA). Again crossing the arterial anastomosis was difficult and had to be carried out in the hybrid operating room. Four months later (20 months after the original procedure), the patient presented with restenosis at the same site. We revised his anastomoses to a more proximal site on the brachial artery: a new arteriovenous anastomosis was performed more proximally on the brachial artery after ligating the vein distal to the Optiflow device and excluding the device. His fistula is still functional at his last follow-up 24 months after his last procedure.

## Discussion

This case report highlights one of the causes of failure of the Optiflow device. As was identified in our patient, post-device stenosis was assumed to be secondary to intimal hyperplasia, and this was probably due to flow dynamics distal to the Optiflow device. There may be other reasons for this stenosis related to device implantation or other host factors that we did not identify. To our knowledge, this is the first case to report Optiflow device stenosis. One lesson learned is the difficulty of crossing these lesions due to device orientation and sharp angles within the vein. Good imaging was needed using a wall-mounted image intensifier, as the smaller portable image intensifier in the outpatient vascular access center did not provide enough resolution to help cross the lesion.

As AV fistulae have become the preferred hemodialysis access in ESRD patients, failure of these AV fistulae has become a significant medical and financial burden.^5^ Several factors have been linked to early fistula failure, including unfavorable hemodynamics leading to stenosis or thrombosis, adverse vessel characteristics and regional anatomy, delayed healing response of the patient, and unsatisfactory surgical technique or inadequate surgical experience in AV fistulae construction. One of the most important causes of AV fistula non-maturation and failure is the early and aggressive occurrence of peri-anastomotic stenosis.^6^ Surgical experience and techniques have been proven to be a major determinant of AV fistula outcome.^4^ A pathological evaluation of several stenotic peri-anastomotic venous segments from failed AV fistulae revealed eccentric intimal hyperplasia and early aggressive medial hypertrophy as the main causes of stenosis.^6^ A novel technique to prevent this complication includes using the Coll-R, which is a sirolimus-eluting collagen sleeve implanted around the graft-vein anastomosis (for arteriovenous grafts) in an attempt to prevent neointimal hyperplasia. It has been shown in a cohort of 12 ESRD patients to improve patency rates with AV grafts.^7^

The Optiflow device was manufactured to address problems associated with inflow failures. First, Optiflow is made of proprietary siliconized polyurethane, which has been extensively used for long-term vascular implants, due to its proven thomboresistant and biocompatibility properties. Also, Optiflow predetermines the angle and area of anastomosis, homogenizing the geometry of the fistula and standardizing the surgical technique. Moreover, by providing a conduit for an end-to-side anastomosis with a fixed outflow area, angle, and geometry calculated according to computational flow modeling, the Optiflow device decreases nonlaminar flow patterns and variations in shear stress across the peri-anastomotic area, protecting the site from neointimal hyperplasia and thus peri-anastomotic venous stenosis.^8^ Porcine studies revealed a significant decrease in stenosis of the venous portion of the AV anastomosis for this device.

The OPEN was a prospective, open-label prospective study that included 41 ESRD patients with these three main inclusion criteria: patients who needed AV fistulae in the upper arm (candidates for lower arm AV fistulae were excluded), inner vein and artery diameters greater than 3 mm, and patients available for a 90-day follow-up.^8^ As mentioned earlier, the main purpose of the Optiflow device was to standardize the surgical technique (vs standard AV fistulae that have significant inter- and intra-operator variability) and provide a fixed area and angle (60°) to provide optimal hemodynamics based on flow modeling, surgeon input, cadaveric testing, and animal studies.

Some studies have reported independent risk factors for early fistula failure, which included obesity, female gender, and African American race.^9–13^ Our patient had none of these characteristics. Despite having a favorable risk profile, and although the Optiflow device was deployed according to the manufacturer’s instructions, our patient developed a 90% stenosis on the venous segment just distal to the Optiflow. This may have been the result of a slight kink in the cephalic vein at the Optiflow connection (although we do not think it was). The OPEN trial has since been terminated, and the Optiflow device has removed from the market for further adjustment of the design.

## Conclusion

This case depicts a failure of the Optiflow device, most likely due to intimal hyperplasia secondary to the flow dynamics distal to the device. Other factors related to device implantation or unidentifiable patient variables might have also contributed to this stenosis. High-resolution imaging was needed to cross this lesion due to device orientation and sharp angles within the vein. To our knowledge, this is the first case to report Optiflow device stenosis.
